# High levels of connectivity over large distances in the diadematid sea urchin *Centrostephanus sylviae*

**DOI:** 10.1371/journal.pone.0259595

**Published:** 2021-11-04

**Authors:** David Veliz, Noemi Rojas-Hernández, Pablo Fibla, Boris Dewitte, Sebastián Cornejo-Guzmán, Carolina Parada

**Affiliations:** 1 Departamento de Ciencias Ecológicas, Facultad de Ciencias, Universidad de Chile, Santiago, Chile; 2 Núcleo Milenio de Ecología y Manejo Sustentable (ESMOI), Coquimbo, Chile; 3 Instituto de Ecología y Biodiversidad (IEB), Santiago, Chile; 4 Centro de Estudios Avanzados en Zonas Áridas (CEAZA), Coquimbo, Chile; 5 Université de Toulouse, CERFACS/CNRS, Toulouse, France; 6 Departamento de Geofísica, Universidad de Concepción, Concepción, Chile; National Cheng Kung University, TAIWAN

## Abstract

Most benthic marine invertebrates with sedentary benthic adult phases have planktonic larvae that permit connectivity between geographically isolated populations. Planktonic larval duration and oceanographic processes are vital to connecting populations of species inhabiting remote and distant islands. In the present study, we analyzed the population genetic structure of the sea urchin *Centrostephanus sylviae*, which inhabits only the Juan Fernández Archipelago and the Desventuradas islands, separated by more than 800 km. For 92 individuals collected from Robinson Crusoe and Selkirk Islands (Juan Fernández Archipelago) and San Ambrosio Island (Desventuradas Islands), 7,067 single nucleotide polymorphisms (SNPs) were obtained. The results did not show a spatial genetic structure for *C*. *sylviae*; relative high migration rates were revealed between the islands. An analysis of the water circulation pattern in the area described a predominant northward water flow with periods of inverted flow, suggesting that larvae could move in both directions. Overall, this evidence suggests that *C*. *sylviae* comprises a single large population composed of individuals separated by more than 800 km.

## Introduction

Knowledge about the genetic structure of populations and gene flow between them is a central issue in population ecology. This information allows us to determine the importance of each population in a metapopulation context and the symmetry of reciprocal migration; key information for species management and conservation plans [1].

Gene flow among populations of benthic marine organisms occurs mainly by the inter-population movement of larvae [2]. The life cycle of most of these organisms includes a low-mobility or sedentary benthic adult phase and a planktonic larval phase [3], the latter of vital importance in the gene flow in fragmented populations [4]. Larval development duration, larval behavior, and oceanographic processes have been described as the main factors affecting larval dispersal of these benthic organisms [5, 6]. It is known that species with long planktonic larvae stages are capable of the largest potential connectivity distance, thereby promoting low population genetic differentiation [7, 8]. Furthermore, oceanographic processes are important in determining the direction of larval movement, in some cases promoting asymmetric gene flow between populations [9–11].

Genetic population variability has been used to study the genetic structure and gene flow among geographically isolated populations. Specifically, the use of thousands of single nucleotide polymorphisms (SNPs) obtained via next-generation sequencing represents a cost-effective alternative to obtain genetic data in non-model organisms [12].

The fragmented nature of the island systems, the span of the larval period and the type and scale of predominant oceanographic processes are all relevant in gene flow of the species inhabiting these islands; larval displacement is fundamental in determining the geographic extension of these populations [13, 14]. Two island systems of Chile separated by 800 km share most of their marine species [15]: the Juan Fernández Archipelago, encompassed by Robinson Crusoe-Santa Clara and Alejandro Selkirk islands, and the Desventuradas Islands, comprised of San Félix and San Ambrosio islands. Due to their high degree of geographic isolation from other areas in the Pacific Ocean, both island systems have a high degree of endemism in both terrestrial and marine biota. The Humboldt Current creates a strong biogeographic barrier between these island systems and the mainland [15]. Porobić et al. (2012) [16] described both mesoscale eddies (connectivity between the islands of the Juan Fernández Archipelago) and meanders (northward flow from the Juan Fernández Archipelago to the Desventuradas Islands) are important oceanographic features in this area, providing physical connectivity and establishing significant particle flow among the different island groups. Previous studies of population connectivity in the Juan Fernández Archipelago and the Desventuradas Islands have only been carried out for the lobster *Jasus frontalis*, which has a larval development of one year [17]. Genetic data and biophysical modeling revealed high connectivity between these island systems for this lobster species [16, 18], suggesting that both systems exhibit high gene flow for a species with a planktonic larval duration of one year.

The sea urchin *Centrostephanus sylviae* Fell 1975 [19] is a species endemic to the Juan Fernández Archipelago and the Desventuradas Islands. There is little published knowledge about the biology and ecology of this species. Friedlander et al. (2016) [15] found higher abundances in the Desventuradas Islands than the Juan Fernández Archipelago. Although the duration of the planktonic development of *C*. *sylviae* is unknown, a closely related species, *C*. *rodgersii*, has a larval development of four months [20]. It has been suggested that this period of larval development allows long distance larval dispersal for *C*. *rodgersii* between Australia and New Zealand [21].

The objective of this study was to estimate the degree of connectivity and population differentiation of the sea urchin *Centrostephanus sylviae* in the Juan Fernández Archipelago and the Desventuradas Islands. Using thousands of SNPs, population genetic structure and the degree of connectivity between populations (migrants between islands) of this species were described. Furthermore, a high-resolution reanalysis model analyzed the climatological currents between the islands to complement the genomic analysis. Considering the previous evidence for *C*. *rodgersii* in Australia and New Zealand, it was expected that the sea urchin *C*. *sylviae* would not show genetic differences between populations in the Juan Fernandez Archipelago and the Desventuradas Islands.

## Materials and methods

### Sample collection

**Fig 1:**
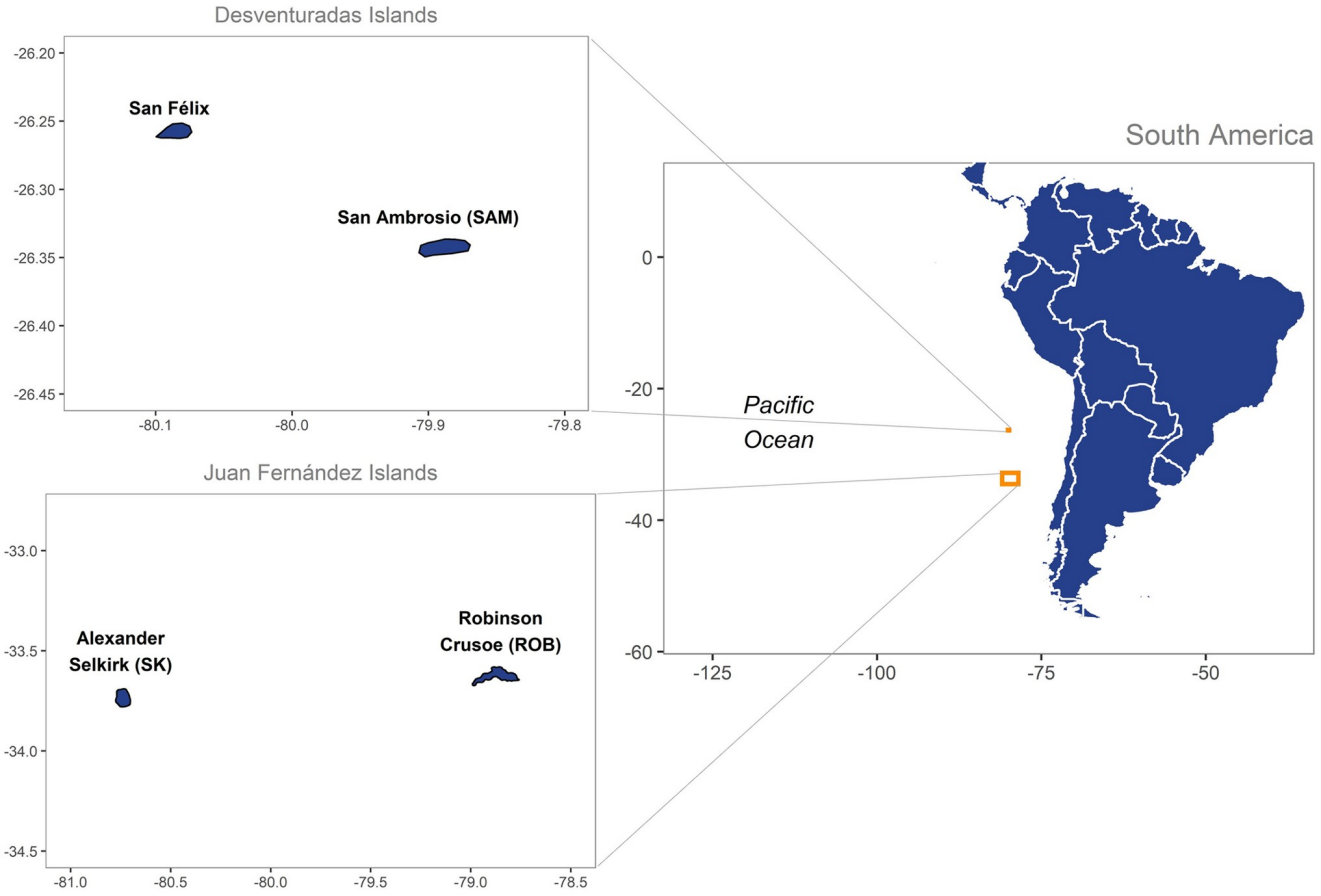
Sampling sites of *Centrostephanus sylviae* in the Juan Fernández Archipelago and Desventuradas Island. Red points represent the sampling areas on each island: Robinson Crusoe (33°36′S, 78°48′W), Alejandro Selkirk (33°48′S, 80°40′W) and San Ambrosio (26°20′S, 79°53′W). Maps drawn using library maps [22] and mapdata [23] implemented in R software [24].

A total of 92 specimens of the sea urchin *Centrostephanus sylviae* were collected from three oceanic islands (Fig 1), 61 of which were collected from the Juan Fernández Archipelago. Of these 61 samples, 29 were collected from Robinson Island (October 2019) and 32 from Selkirk Island (December 2019). In addition, 31 individuals were collected from San Ambrosio Island of the Desventuradas Islands in January 2020. Samples were obtained by scuba diving in shallow water. The Aristotle’s lantern of each specimen was preserved in 95% alcohol for genetic analysis. Sampling was performed under Fondecyt grant 1191606 and the Monitoring Program for Crustacean Fisheries of the Juan Fernández Archipelago (IFOP grant 2019) and sampling permission was granted by the Port Captain at Juan Fernández Archipelago, Chile.

### Genetic analysis

A small piece of muscle collected from each Aristotle’s lantern was placed in 96-well PCR plates with 80 μL of 99% ethanol for genetic analysis. DNA extraction and massive sequencing were performed at Dart Diversity Arrays Technology Pty Ltd., Canberra, Australia (www.diversityarrays.com). DNA was digested with the restriction enzymes Sbfl and Pstl following Kilian et al. (2012) [25]. Fragments > 200 bp were ligated with an 8 base pair barcode and amplified by PCR. The PCR products were standardized and sequenced on a HiSeq 2500 (Illumina Inc, San Diego, USA).

### Quality control and initial SNP calling

Dart Diversity’s bioinformatics service performed demultiplexing and removal of the DNA barcodes. More information about the detection of SNPs is described in Kilian et al. (2012). Raw SNP data from Dart were filtered using the dartR library [26] implemented in the R program [24]. This filter eliminated i) only one SNPs was retained in the reads containing two or more SNPs, ii) loci with a read depth below five or above 200, iii) loci with < 99% reproducibility, iv) monomorphic loci, v) loci with > 10% missing data, vi) individuals with > 5% missing data and vii) all SNPs with a minimum allele frequency (MAF) < 1%.

To avoid bias in estimating the differences between populations, the loci identified as under selection were eliminated. Four different approaches were used for this: i) the method based on likelihood implemented in the outflank function of the dartR library, ii) the method based on principal components, implemented in the PCAdapt library [27], also implemented in the R software, iii) the Bayesian method implemented in the BayeScan program [28], and iv) the method based on the relationship between F_ST_ and heterozygosity implemented in the Fsthet library [29] of the R software. In addition, all loci that showed evidence of selection in at least 2/4 methods implemented were removed. Finally, loci with significant departures from Hardy-Weinberg equilibrium in all sampling sites were removed using the dartR library, and loci showing linkage disequilibrium > 0.5 in all sampling sites were filtered with PLINK 2.0 software [30].

### Genetic diversity

Expected heterozygosity (Hexp.), observed heterozygosity (Hobs.), and the inbreeding coefficient (F_IS_) were estimated using GENETIX v 4.05 [31]. Allelic richness (AR) was estimated with the divBasic function of the diveRsity library [32].

### Population genetic structure

Population genetic structure was estimated using the SNP database for the three sampling sites of *C*. *sylviae*. Three methods were used for this: i) A principal coordinates analysis (PCoA) was used to observe the distribution of individuals in a multivariate space qualitatively, using the dartR library implemented in the software R; ii) the paired F_ST_ index was assessed with the gl.fst.pop function implemented in the dartR library; iii) the most probable number of genetic clusters (K) was estimated using the Bayesian approach implemented in the Structure software [33]. The admixture model and correlation of allele frequencies were used as input. The procedure was performed three times for each K (from K = 1 to K = 4) with a burn-in of 100,000 iterations and an after-burn-in of 200,000 iterations. The most likely number of clusters was estimated using the output in the Structure Harvester program [34]. The probability of each K was estimated using the method described in the Structure manual [35].

Finally, the direction and magnitude of the gene flow of *C*. *sylviae* between the islands were estimated using the divMigrate function of the diveRsity library [32] implemented in the R software. The effective number of migrants (Nm) was used as a distance measurement, and the asymmetry of the flow between the islands was tested using a bootstrap of 1000 iterations.

### Oceanographic circulation in the area

GLOBAGLOBAL_REANALYSIS_PHY_001_030 (CGLOPHY030) and CMEMSGLOBAL_ANALYSIS_FORECAST_PHY_001_024 (CGLOPHY024) products distributed by CMEMS (http://marine.copernicus.eu/) were used to understand circulation patterns between the Juan Fernandez Archipelago and the Desventuradas Islands. Briefly, CGLOPHY030 is a daily global reanalysis model [36] providing resolution and depths, with output spanning between 1993 to 2018, and GLOPHY024 is a daily global forecast model [37] of a high-resolution regular grid 1/12° (~ 8 km) and 50 depth levels (from surface to 5500 m). The model output spanned from January 2016 to the present. It is based on the hydrodynamic model of the ocean Nucleus for European Modelling of the Ocean (NEMO) [38] that assimilates altimetry, sea surface temperature satellite data, vertical temperature, and salinity. NEMO v3.1 was used in our study.

Both model outputs were used to characterize the meridional and longitudinal velocities and transport patterns between the Juan Fernandez Archipelago and the Desventuradas Islands from 1993 to 2020. One-hundred meters of averaged U and V velocity components were extracted in the region between 81°–78°W and 35°–25.5°S that includes both island groups. Seasonal spatial climatology of the meridional and longitudinal velocities was also estimated. A time series of meridional and longitudinal velocities and transport between regions, averaged for the first 100 m and between 1000 and 100 m, was estimated for the same period (1993–2020).

## Results

In the 92 individuals analyzed, 85,933 raw SNPs were obtained. After applying filters and removing outlier loci, 7,067 SNPs and 89 individuals were retained: 28 from Robinson, 31 from Selkirk, and 30 from San Ambrosio. The allelic richness and heterozygosity of these filtered data showed similar values among the three islands studied (Table 1).

**Table 1:** Summary of data used in the analysis of the sea urchin *C*. *sylviae* including sample size (n), allelic richness (AR) observed heterozygosity (Hobs.), expected heterozygosity (Hexp.), non-biased heterozygosity (Hn.b.) and F_IS_ at each study site.

Site	n	AR	Hobs.	Hexp.	Hn.b.	F_IS_
Robinson	28	1.71	0.1095	0.1466	0.1494	0.2709
Selkirk	31	1.72	0.1107	0.1470	0.1495	0.26288
San Ambrosio	30	1.7	0.1024	0.1431	0.1456	0.30043

**Fig 2:**
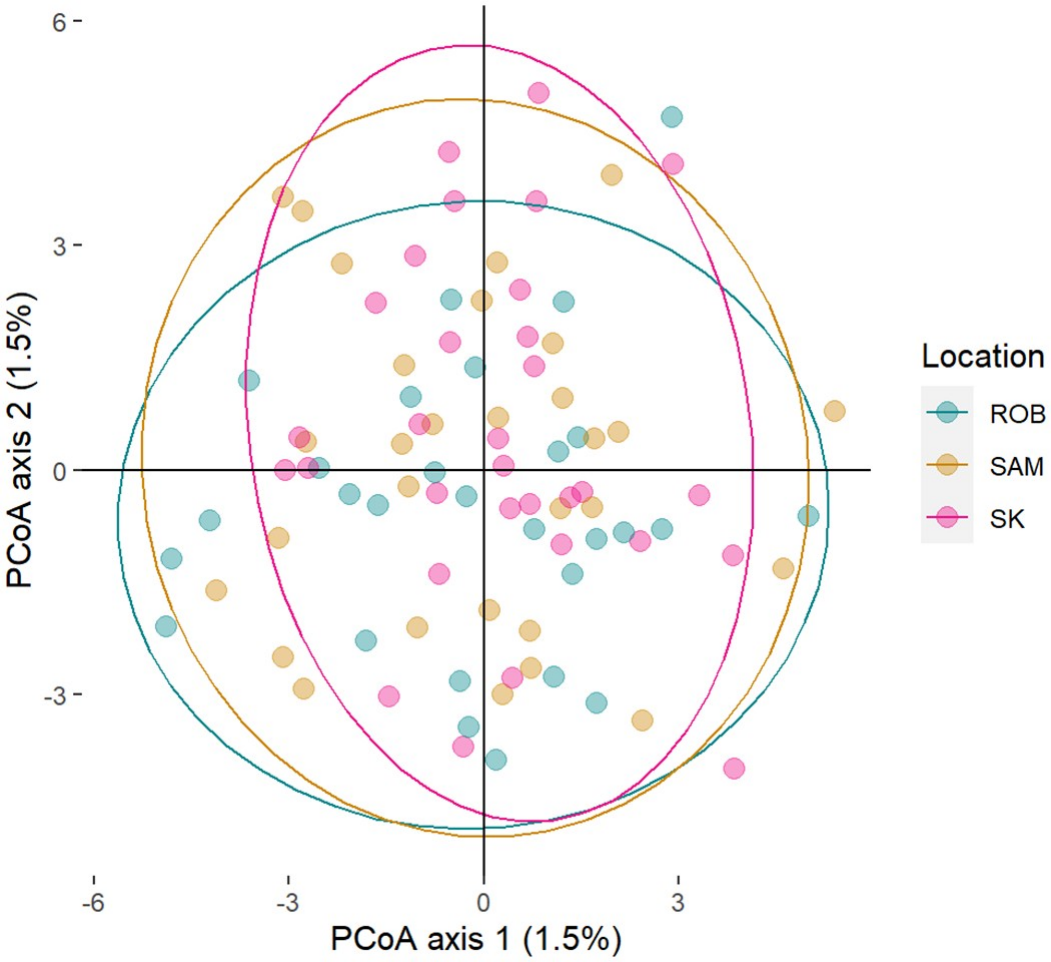
Principal coordinate analysis (PCoA) performed with sea urchin *C*. *sylviae* data obtained from Robinson Island (ROB), San Ambrosio Island (SAM), and Selkirk Island (SK).

**Fig 3:**
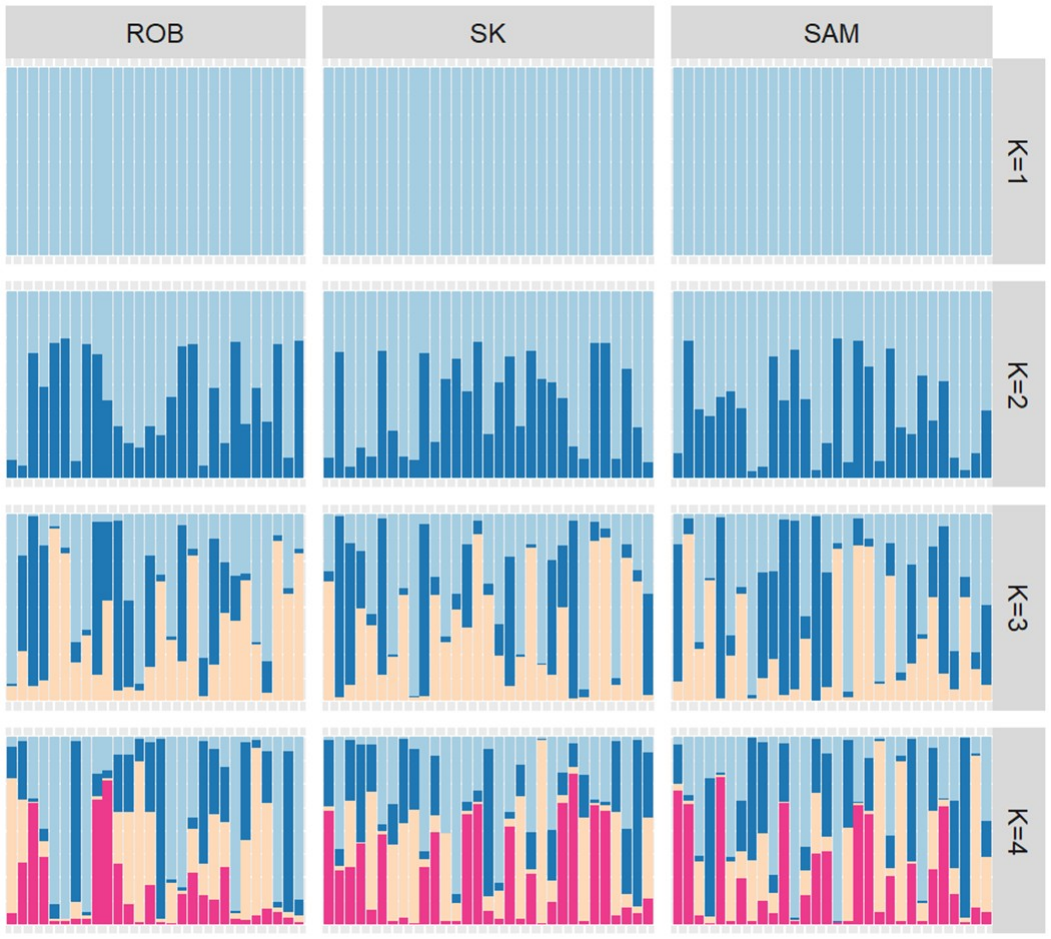
Population structure of the sea urchin *C*. *sylviae* inferred using the software STRUCTURE for K = 1 to K = 4 of 89 individuals from the three studied islands. A vertical line represents each individual, and each color represents the probability of belonging to one of the genetic clusters. ROB = Robinson Island, SAM = San Ambrosio Island, and SK = Selkirk Island.

**Fig 4:**
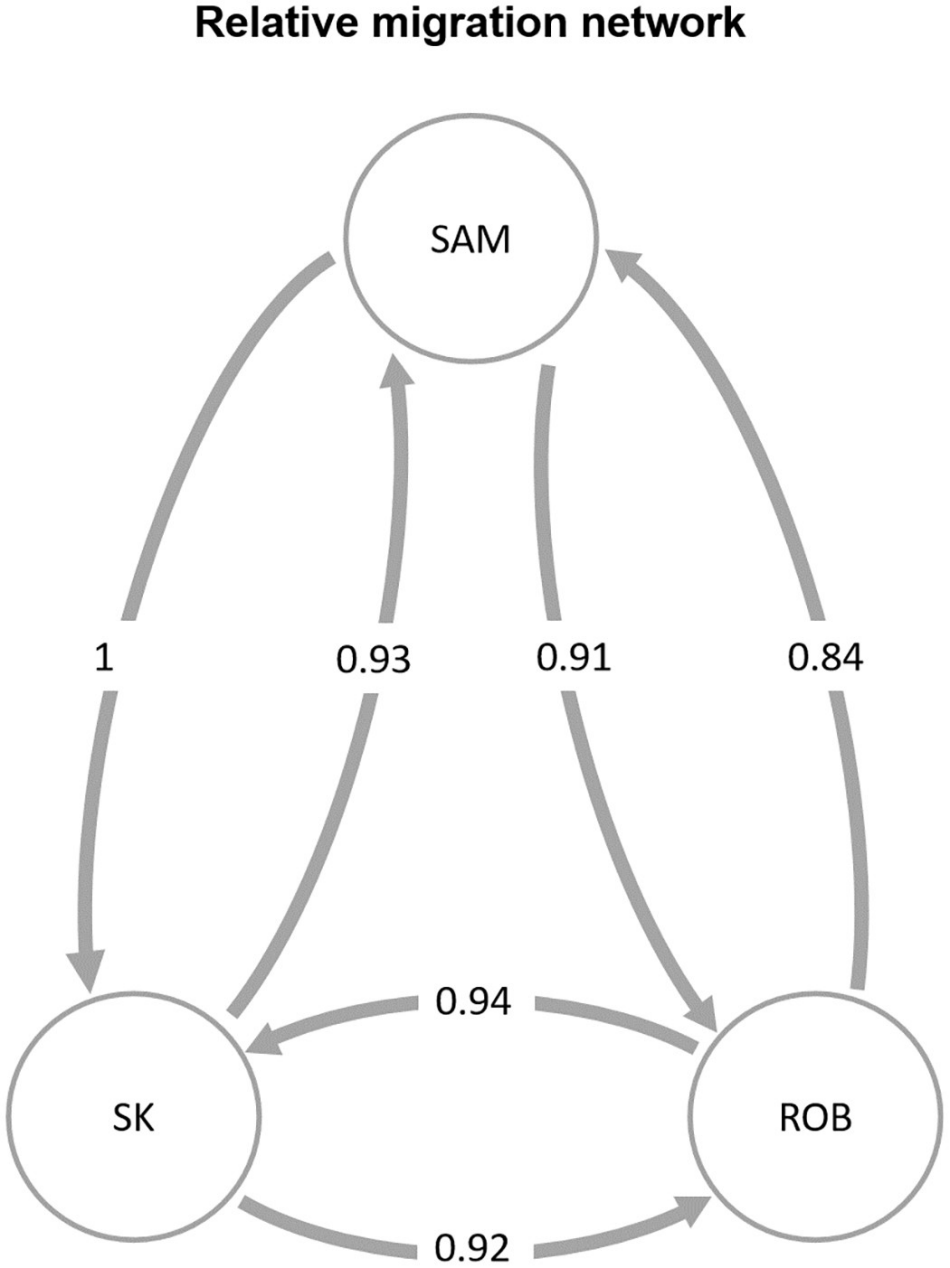
Migration network for the sampling sites of *C*. *sylviae* on Robinson Island (ROB), Selkirk Island (SK), and Sam Ambrosio Island (SAM). This figure was generated with the divMigrate function implemented in diveRsity (Keenan et al., 2013 [32]). Each vertex represents a sampling site, and each arrow represents the magnitude and direction of the relative migration.

Population genetic structure analysis did not detect significant differences among the studied sites. The PCoA showed an overlap of the individuals in the multivariate space, suggesting no segregation of the populations (Fig 2). Significant differences in F_ST_ were not detected between the pairs of sites compared (Table 2). The Bayesian analysis implemented in the Structure software showed that K = 1 had the lowest value of ln (K) (mean LNP [K] = -365659.3, p = 0.999). However, Evanno’s method indicated K = 3 as the most likely number of clusters. The graphs from K = 1 to K = 4 (Fig 3) suggest no clear genetic separation between the islands; thus, K = 1 is the most biologically viable explanation. Finally, the analysis performed with divMigrate suggests strong gene flow between the islands (Fig 4); the bootstrap did not detect significant asymmetric gene flow between the islands (p > 0.05).

**Table 2:** Pairwise F_ST_ (above diagonal) and P-value (below diagonal) values for individuals collected in the islands studied.

	Robinson	Selkirk	San Ambrosio
Robinson		-0.00011	0.00056
Selkirk	0.609		0.00003
San Ambrosio	0.139	0.476	

**Fig 5:**
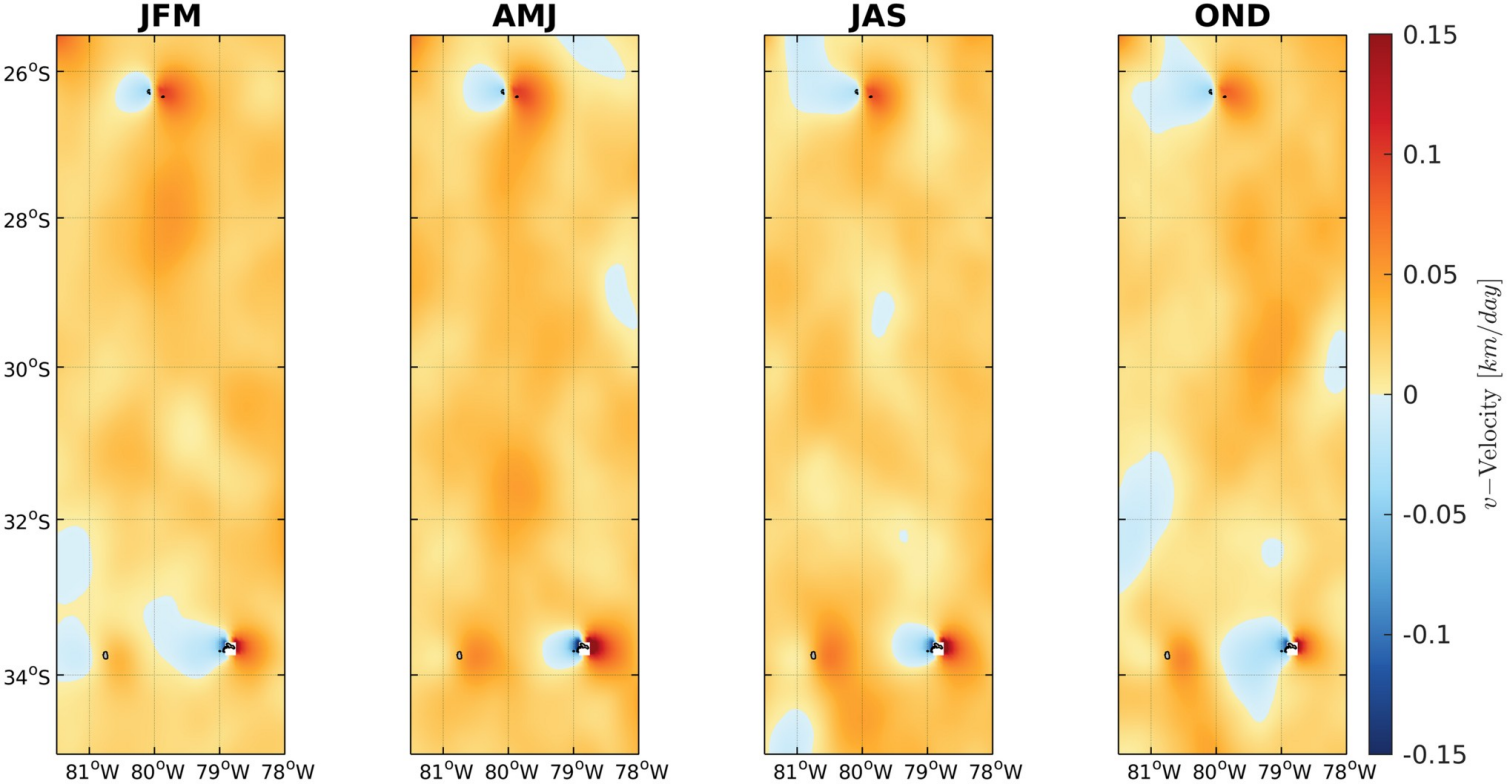
Seasonal (JFM, summer; AMJ, fall; JAS, winter; OND, spring) meridional (positive, northward; negative, southward) velocities averaged over the first 100 m between Juan Fernandez Archipelago and the Desventuradas Islands, 25.5–35°S and 81.5–78°W. Data was obtained with Copernicus model products freely distributed by CMEMS (http://marine.copernicus.eu/).

**Fig 6:**
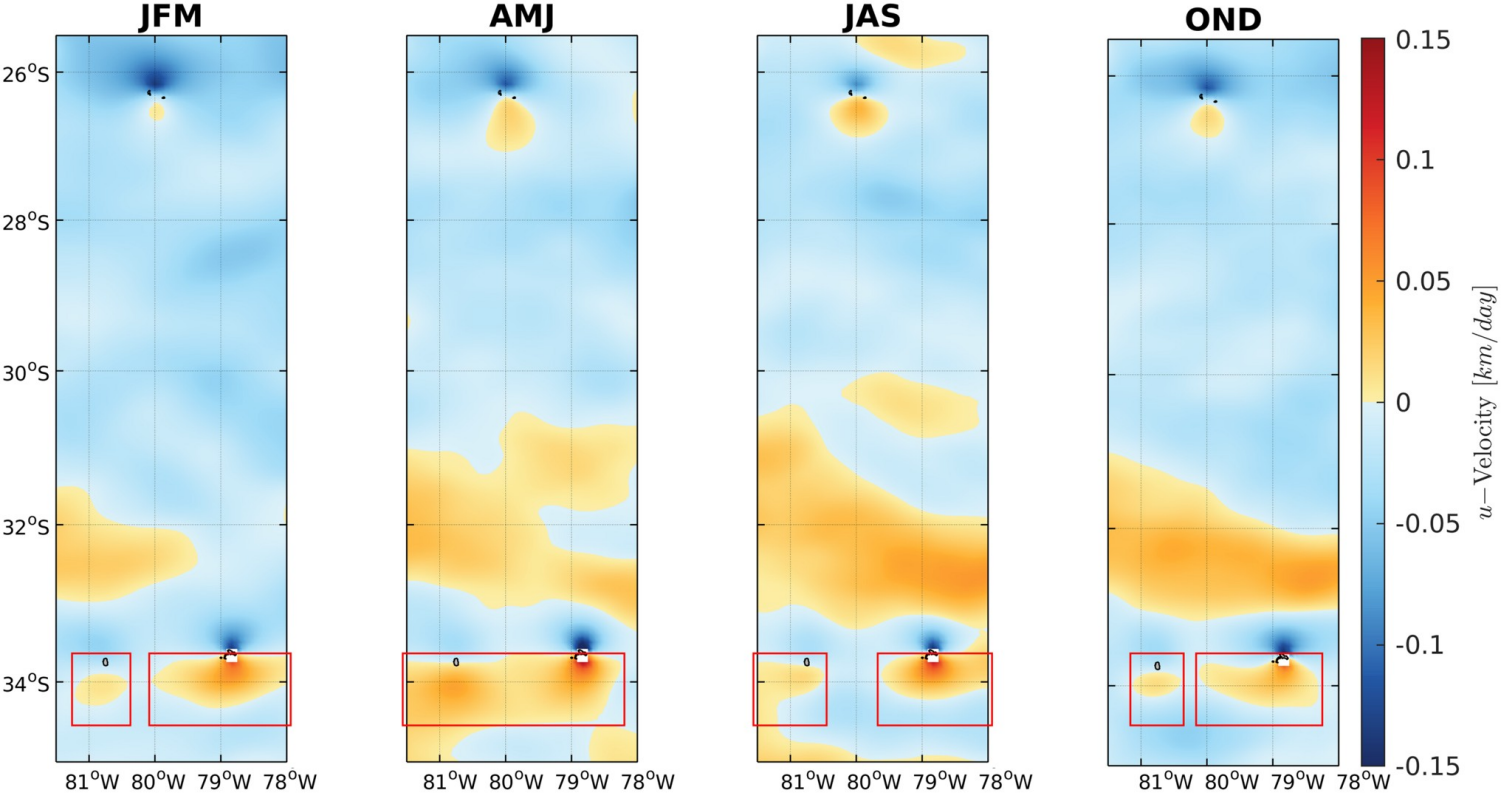
Seasonal (JFM, summer; AMJ, fall; JAS, winter; OND, spring) longitudinal (positive, westward; negative, eastward) velocities averaged over the first 100 m in the Juan Fernandez Archipelago and the Desventuradas Islands, 25.5–35°S and 81.5–78°W. Data was obtained with Copernicus model products freely distributed by CMEMS (http://marine.copernicus.eu/).

No seasonal changes were observed at the meridional velocities, with a predominance of northward velocities between the region of the Juan Fernandez Archipelago and the Desventuradas Islands (Fig 5). Southward velocities were not significant. Within the Juan Fernandez Archipelago, an eastward flow (light blue) between Selkirk and Robinson islands characterized the velocities during all seasons (see the flow inside the black squares) except for fall, when the flow associated to the southward region of the islands reversed to westward velocities (oranges) connecting Robinson towards Selkirk islands (Fig 6).

**Fig 7:**
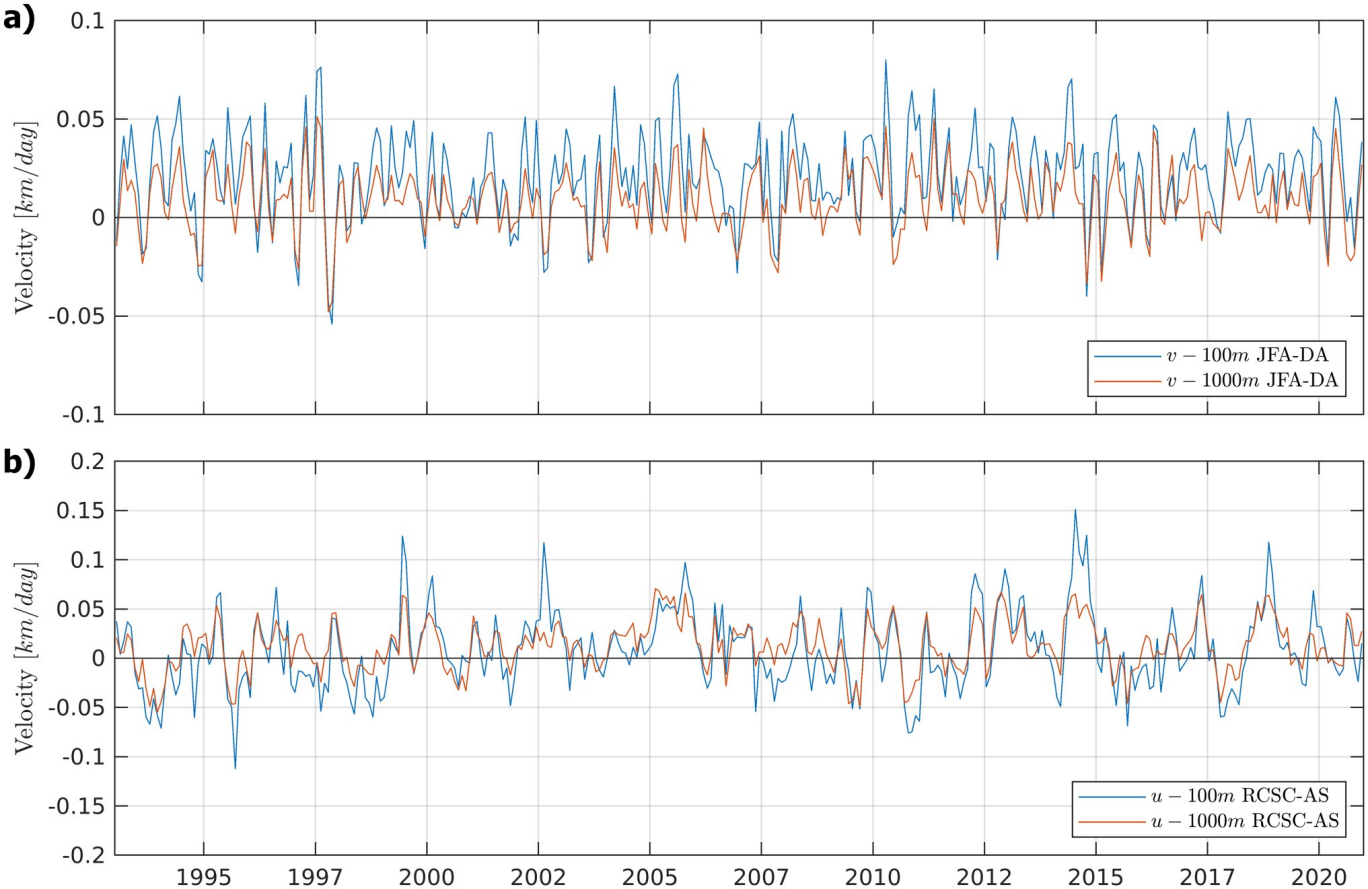
a) Monthly meridional mean velocity (blue line, mean 0–100 m; red line, mean 100–1000 m) time series for the region 25.5–35°S and 81.5–78°W (positive, northward; negative, southward) explaining connectivity between Juan Fernandez Archilepago (JFA) and Desventuradas Islands (DA); b) Monthly longitudinal mean velocity (blue line, mean 0–100 m; red line, mean 100–1000 m) time series (positive, eastward; negative, westward) explaining connectivity between Robinson and Selkirk islands. Data was obtained with Copernicus model products freely distributed by CMEMS (http://marine.copernicus.eu/).

Monthly meridional velocity variability time series showed a northward predominance between Juan Fernandez and Desventuradas in the studied years (1993 to 2020) in the surface depth (mean 0–100 m) and the deep strata (100–1000 m), with brief periods of inverted flows (southward). Monthly longitudinal velocity time series showed eastward and westward flow connecting Selkirk and Robinson islands (Fig 7), with a similar current pattern in the surface and deep strata.

## Discussion

As expected, our results suggest that the sea urchin *Centrostephanus sylviae* inhabiting the Desventuradas Islands and the Juan Fernández Archipelago are a single panmictic genetic population with strong gene flow between these two systems, even though they are separated by more than 800 km. Since adult echinoids have low mobility, migration and gene flow occur mainly via planktonic larvae.

Previous evidence showed high dispersal of larvae in sea urchins, which allows their populations to cover large geographic areas. For example, *C*. *rodgersii* shows weak genetic differentiation and no isolation over thousands of kilometers between eastern Australia and northern New Zealand [21]. Other sea urchins have similarly shown no large-scale genetic differences: *Strongylocentrotus droebachiensis* inhabiting Atlantic Canada [39], *Paracentrotus lividus* in the Mediterranean [40], *Tripneustes gratilla* in the Philippines [41], and *S*. *franciscanus* in California [42], among others.

Other benthic invertebrate taxa (e.g., marine crabs) with similar planktonic larval duration have large geographic populations, suggesting high dispersal of the larvae. For example, *Cancer pagurus* have a planktonic larval duration of 3 months [43] and did not show genetic differences among populations sampled in the Swedish Sea [44]. Moreover, Rojas-Hernández et al. (2016) [45] did not detect differences along 700 km of coastline in Chile in the commercial crab *Metacarcinus edwardsii*, which has a planktonic development of 60 days (Quintana, 1989) [46]. In general, the evidence of sea urchins and crabs suggests that a planktonic development of at least 60 days allows high dispersion between geographically remote locations.

In the same studied islands, Porobic et al. (2013) [18] described the population genetic structure of the lobster *Jasus frontalis*. In this study, the individuals sampled in the Juan Fernández Archipelago and Desventuradas Islands systems showed a single panmictic population and high gene flow between islands, revealed by COI gene variability. This evidence is consistent with the pattern detected in the current study. Furthermore, Porobic et al. (2012) [16] used a biophysical model to study the connectivity of *J*. *frontalis* populations between the Juan Fernández Archipelago and the Desventuradas Islands. The authors detected an asymmetric connectivity pattern between these islands, consisting of significantly greater larvae dispersal from Juan Fernández to the Desventuradas Islands. Our analysis using Nemo corroborates the analysis performed by Porobic et al. (2012) [16] that used the hydrodynamic model OFES (Ocean Model for the Earth Simulator) described by Masumoto et al. (2004) [47]. The velocity field and time-series analyses in the present study support this asymmetric relationship in the circulation pattern, revealing a predominance of northward velocities connecting the Juan Fernandez system to the Desventuradas Islands. The genetic analysis of *C*. *sylviae* did not show an asymmetric gene flow pattern as expected based on the biophysical model, suggesting that other factors could be involved in promoting the high gene flow observed in the current study. Therefore, it is necessary to continue studying dispersal patterns in this area to understand how the larval behavior, circulation (both mean and turbulent flow) and the biogeochemical environment relate to species connectivity between these two island systems, since it is known that the crustacean (e.g., [48]) and sea urchin larvae [49] have larval behavior of vertical migration. It was described that mean eddy activity corresponding to transient small scale (~10 to 100 km) recirculation patterns (eddies) is at a relative maximum between the two island systems, which could selectively affect the dispersion of species with planktonic larvae. Furthermore, Desventuradas and Juan Fernandez are in the neighborhood of an intense oxygen minimum zone [50], so that sharp oxygen gradients could also influence the species in a distinctive way.

Finally, *C*. *sylvae* larvae could move from these islands separated by more than 800 km and continue to connect these geographic groups present on the different islands studied. This evidence is crucial to understand how species are connected in island systems that have marine protected areas. In 2015, Chile created the Nazca-Desventuradas Marine Park of approximately 300,000 km^2^ [51] which includes the Desventuradas Islands (San Ambrosio and San Felix islands), with the main goal being the conservation of the biological processes in the area. It is important to note that the Desventuradas Islands and Robinson Crusoe Island (Juan Fernandez Archipelago) are uninhabited and inhabited by humans, respectively. Thus, high connectivity suggests that species exploited on the inhabited island (Robinson Crusoe Island) could be currently subsidized from this Marine Park. New investigations of species with different planktonic larval durations may help determine the level of connectivity of the benthic marine communities shared between these two island systems and, incidentally, may elucidate the role of the marine protected area in the face of exploitation pressure in the area.
